# Earth stewardship: Shaping a sustainable future through interacting policy and norm shifts

**DOI:** 10.1007/s13280-022-01721-3

**Published:** 2022-04-05

**Authors:** F. Stuart Chapin, Elke U. Weber, Elena M. Bennett, Reinette Biggs, Jeroen van den Bergh, W. Neil Adger, Anne-Sophie Crépin, Stephen Polasky, Carl Folke, Marten Scheffer, Kathleen Segerson, John M. Anderies, Scott Barrett, Juan-Camilo Cardenas, Stephen R. Carpenter, Joern Fischer, Nils Kautsky, Simon A. Levin, Jason F. Shogren, Brian Walker, James Wilen, Aart de Zeeuw

**Affiliations:** 1 Professor Emeritus, Institute of Arctic Biology, University of Alaska Fairbanks, Fairbanks, AK 99775 USA; 2 Andlinger Center, Princeton University, Princeton, NJ 08544 USA; 3 Bieler School of Environment, McGill University, Ste. Anne de Bellevue, Quebec, H9X 3V9 Canada; 4 Centre for Sustainability Transitions, Stellenbosch University, Stellenbosch, South Africa; 5 Stockholm Resilience Centre, Stockholm University, 104 05 Stockholm, Sweden; 6 ICTA, Universitat Autònoma de Barcelona, 08193 Bellaterra, Spain; 7 SBE & IVM, Vrije Universiteit Amsterdam, 1081 HV Amsterdam, The Netherlands; 8 College of Life and Environmental Sciences, University of Exeter, Exeter, EX4 4RJ UK; 9 Beijer Institute of Ecological Economics, Royal Swedish Academy of Sciences, 104 05 Stockholm, Sweden; 10 Department of Applied Economics, University of Minnesota, St. Paul, MN 55108 USA; 11 Stockholm Resilience Centre, Stockholm University, Stockholm, Sweden; 12 Department of Environmental Sciences, Wageningen University, 6700 AA Wageningen, The Netherlands; 13 Department of Economics, University of Connecticut, Storrs, CT 06269-1063 USA; 14 School of Human Evolution and Social Change and School of Sustainability, Arizona State University, Tempe, AZ 85287-2401 USA; 15 Earth Institute, Columbia University, New York, NY 10027 USA; 16 Department of Economics, University of Massachusetts Amherst, Amherst, MA 01002 USA; 17 Center for Limnology, University of Wisconsin, Madison, WI 53706-1413 USA; 18 Faculty of Sustainability, Leuphana Universität Lüneburg, 21335 Lüneburg, Germany; 19 Professor Emeritus, Department of Ecology, Environment and Plant Sciences, Stockholm University, 106 91 Stockholm, Sweden; 20 Department of Ecology and Evolutionary Biology, Princeton University, Princeton, NJ 08544-1003 USA; 21 Department of Economics, University of Wyoming, Laramie, WY 82071-3985 USA; 22 CSIRO Land and Water, Canberra, ACT 2601 Australia; 23 Department of Agriculture and Resource Economics, University of California, Davis, Davis, CA 95616 USA; 24 Tilburg School of Economics and Management, 5000 LE Tilburg, The Netherlands

**Keywords:** Anthropocene, Earth stewardship, Institutions, Market economy, Social norms, Transformation

## Abstract

Transformation toward a sustainable future requires an earth stewardship approach to shift society from its current goal of increasing material wealth to a vision of sustaining built, natural, human, and social capital—equitably distributed across society, within and among nations. Widespread concern about earth’s current trajectory and support for actions that would foster more sustainable pathways suggests potential social tipping points in public demand for an earth stewardship vision. Here, we draw on empirical studies and theory to show that movement toward a stewardship vision can be facilitated by changes in either policy incentives or social norms. Our novel contribution is to point out that both norms and incentives must change and can do so interactively. This can be facilitated through leverage points and complementarities across policy areas, based on values, system design, and agency. Potential catalysts include novel democratic institutions and engagement of non-governmental actors, such as businesses, civic leaders, and social movements as agents for redistribution of power. Because no single intervention will transform the world, a key challenge is to align actions to be synergistic, persistent, and scalable.

## Introduction

Nature and society stand at a critical juncture. Rates of climate change, environmental degradation, and levels of social inequality are rising (IPCC 2018; Díaz et al. 2019). However, society has been unsuccessful in transforming toward more sustainable pathways. The purpose of this paper is to outline a vision and a new pragmatic approach to transform toward sustainability. This approach focuses on interactions, synergies, and alignment between incentives and social norms to shape a more sustainable future for society and the biosphere.

A broad body of recent sustainability research and theory has identified many factors that inform practical stewardship strategies (e.g., Bennett et al. 2016; Loorbach et al. 2017; O’Brien 2018; Díaz et al. 2019; Clark and Harley 2020; Folke et al. 2021). These conclude the following: (1) Transformation requires fundamental changes, rather than minor tweaks, to restore a sustainable relationship between society and nature as part of the biosphere. (2) Transformation results from interacting changes in many elements through multiple processes. (3) The resulting complexity creates high uncertainty in outcomes, requiring responses to be resilient to unforeseen circumstances. (4) Transformation creates both winners and losers, requiring careful attention to equitable distribution of resources and redistribution of power to shape change. Building on these insights, we identify a suite of approaches that are grounded in theory, sensitive to context, and provide practical guidance to support transformative change at local-to-global scales.

We define earth stewardship as the proactive shaping of physical, biological, and social conditions to sustain, rather than disrupt, critical earth-system processes in support of nature and human wellbeing at local-to-planetary scales (Chapin et al. 2010; Steffen et al. 2011). Its key policy-relevant element is prioritization of approaches that shape sustainable and equitable future changes rather than return to some prior system state. This approach recognizes that society, as part of, and dependent on, a resilient biosphere, affects and responds to many interacting earth-system processes and globally networked risks (Galaz et al. 2017), including climate-driven changes in water and food supply, increased frequency and severity of natural disasters, sea level rise, loss of ecosystem services (e.g., water filtration, pollination, and flood control), and disease risk due to antibiotic resistance, changing wildlife habitat, and increasing mobility. Motivations for stewardship include responsibility to care for vulnerable people, species, and ecosystems (Enqvist et al. 2018), concern for the safety of others and of one’s community and nation (Brown et al. 2019), and the values that link people with nature (Klain et al. 2017). Stewardship has a temporal dimension encompassing care for the future; an environmental dimension entailing care for the earth and other species; and an equity dimension focusing on a fairer distribution of resources, rights, responsibilities, and power across society, within and among nations.

In this paper, we emphasize approaches that can be initiated now by all countries, but need to be reflective of their current and historical responsibilities and uneven access to power and resources to implement potential solutions between elements of society. The underlying principles are that all countries and sections of society should have the opportunities, resources, and capacity to develop stewardship approaches that meet their goals and needs to ensure that all people have equitable access and power to develop sustainably.

## Leverage points for operationalizing earth stewardship

Stewardship-based transformation involves change toward a new system with fundamentally different human–environment interactions and feedbacks (Westley et al. 2011). Both planned changes and unanticipated events contribute to transformation. The planned components of transformation require a vision of desired outcomes and an approach to implementing that vision (Olsson et al. 2006; Bennett et al. 2016).

One reason for society’s limited success in transforming toward sustainability in many countries is the predominant focus on specific policies with limited breadth of impact. Meadows (1999) and Abson et al. (2017) identified several categories of interventions (leverage points) that differ in feasibility and potential impact. They argued that the greatest potential for deep systemic change comes from a shift in the vision that guides societal goals. In contrast, more modest leverage points, such as specific policies, may be easier to implement and enforce but are unlikely to cause broad systemic change. O’Brien (2018) emphasized that adaptive challenges (e.g., addressing social equity or climate change) require both a new way of viewing problems (i.e., vision) and solution-focused policies that target implementation.

**Fig. 1:**
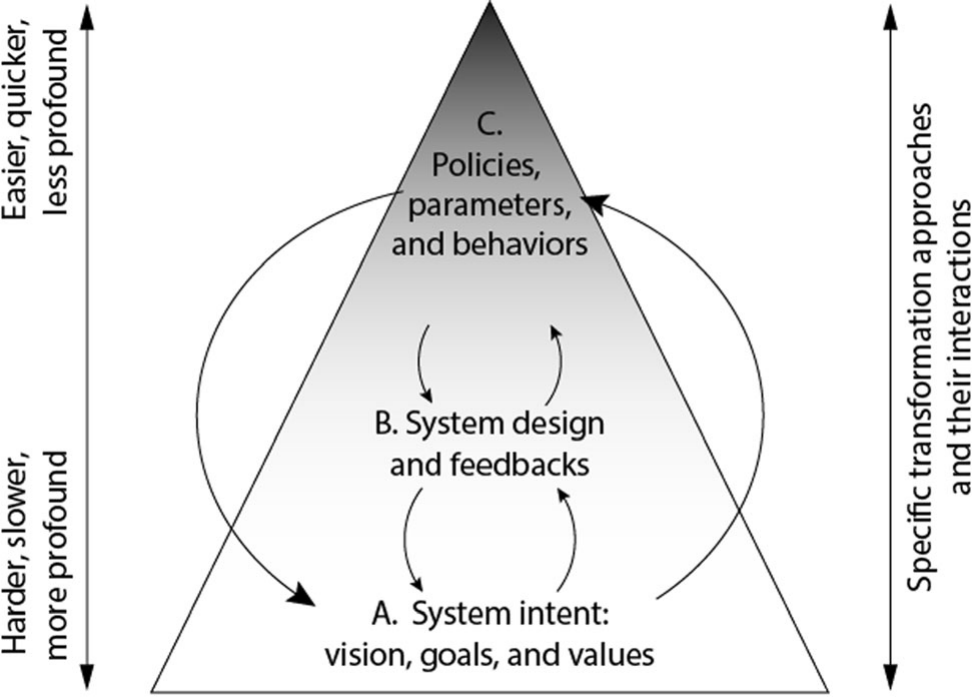
Leverage points for transformation, based on Abson et al. (2017) and O’Brien (2018). Arrows indicate interactions among categories of leverage points. See Table 1 and accompanying text for examples of specific transformation approaches

In this paper, we bridge these frameworks by focusing on the integration and interactions among multiple leverage points. Our approach is to provide (1) a systemic vision to guide choices of pathways toward sustainable and equitable change, (2) identification of feedbacks and design features (e.g., norms and incentives) that can be shaped to align desired changes among multiple system components, and (3) examples of policies, institutions, and novel actors that might trigger or support desired changes (Fig. 1). Our novel contribution is a focus on linkages, complementarity, and alignment among leverage points to maximize their effectiveness.

We describe five pragmatic strategy elements to trigger transformation (see Table 1 for a numbered overview) drawing on studies such as “Seeds of good Anthropocenes,” which synthesized 100 bottom-up studies of transformative change (Bennett et al. 2016; Pereira et al. 2018). This synthesis, which now incorporates 500 studies, illustrates interactions among vision, system dynamics, and initiating triggers. For example, depopulation of agricultural landscapes in rural Japan had reduced the diversity and productivity of food crops. One *vision* for restoring these landscapes was to rejuvenate Satoyama traditional agriculture that integrated terraced rice paddies with a diversity of food crops and other cultural features (Takeuchi et al. 2016). A scenario (pathway) to accomplish this vision was to draw on a growing interest among urban residents to learn about and participate in traditional food production. The trigger that enabled this to occur was a program of homestays for urban residents who provided financial support and volunteer labor for the agricultural initiative. Although the long-term sustainability of this initiative cannot be predicted, the pathway met agricultural and cultural goals that were unavailable in the depopulated landscape that it replaced.

**Table 1:** Leverage points for operationalizing an earth stewardship approach, with details provided in sections A, B, and C

Transformation approaches	Illustrative goals and actions
A. Vision, goals, and values	
1. Change vision and goals	Identify a set of broadly accepted goals and values that, if acted upon, would reduce consumption-driven changes in climate and ecosystems that currently threaten the wellbeing of people and nature
B. System design and feedbacks	
2. Shift social norms and behavior	Shift social norms to foster less-consumptive behaviors in order to contribute to sustainable wellbeing of people and nature within and among nations
3. Incentivize sustainable production/consumption decisions	Internalize environmental and social costs in market economies to support societal needs for built, natural, human, and social capital
C. Agency to influence institutions and policies	
4. Engage influential actors	Engage businesses, NGOs, civic leaders, and social movements to support sustainability goals and foster social–ecological resilience
5. Foster deliberative democracy	Design new institutions, such as citizen assemblies, that foster prosocial change and constrain the power of forces that resist such changes

The abolition of slavery in Britain illustrates how interactions among multiple leverage points can contribute to systemwide changes (Hochschild 2005). In 1787, twelve influential religious men began to advocate a vision of the moral unacceptability of slavery (A1 in Table 1). Over 46 years, multiple processes (public education, boycotts, and activist campaigns) shifted societal norms to the point that slavery and slave-produced goods were deemed “unacceptable” (B2). Development of a sugar-beet industry in Europe and financial compensation to slave-owners after slavery abolition shifted economic incentives away from slavery as an institution necessary for sugar production (B3). Local anti-slavery committees in individual British towns coordinated efforts and engaged parliamentarians (C5). Lord Wilberforce recruited colleagues and built a political case that led to legislation prohibiting the trading of slaves within the British Empire in 1807 and abolishing slavery altogether in 1833 (C4 and C5). It took another 55 years until the last country (Brazil) abolished slavery. It is doubtful that any one of these campaign strategies or unforeseen events would have single-handedly transformed Britain and its colonies away from slavery, but the application of multiple strategies focused on multiple interacting leverage points contributed to this paradigm-shifting transformation. These interventions included advocacy of a different paradigm, shifting of social norms and behavior, and policy interventions that changed economics, laws, and norms.

Next, we discuss items A to C of Table 1 in more detail.

### A: Vision, goals, and values

Vision, goals, and values represent the deepest leverage and relate to the intent and target of earth stewardship (what society *should* do).

#### A1: Change vision and goals

Our guiding vision is that sustainable earth processes (both social and biophysical) are a prerequisite for wellbeing of people and the rest of nature. This system perspective aligns with key intents of most major religions and indigenous worldviews—for example, to care for the earth and vulnerable people (Pope Francis 2015; UN Environment Programme 2021). To address this intent, institutions (e.g., religions, non-governmental organizations [NGOs], social movements, businesses, and government) should advocate actions that enhance the wellbeing of people and nature by halting climate change and ecosystem degradation and by reducing social inequity. This vision would be fostered if society’s goals expanded beyond provision of material goods and private wealth, which characterized the policies of many nations in the nineteenth and twentieth centuries, to a commitment to ensure the equitable wellbeing of current and future generations of humanity and other species and the persistence of their life-support systems (Trebeck and Williams 2019). In the absence of a long-term guiding vision for sustainability, short-term vested interests and concerns will likely continue to predominate, and humanity is unlikely to effectively address planetary degradation.

Aiming for continuous economic growth is a widespread paradigm that currently guides many governmental, business, and personal decisions. This paradigm has contributed to environmental degradation and inequality by accelerating resource extraction and use by those who can afford it (Escobar 2015; Dasgupta 2021). If the concept of wealth were broadened from material goods (built capital) to include natural capital (natural resources and ecosystem services), human capital (the capacity of people to accomplish their goals through health, education, and training) and social capital (empowerment for collective action) equitably distributed across society, this would provide a more sustainable social-ecological foundation for human wellbeing (Polasky et al. 2015; Clark and Harley 2020). Enhancing human and social capital, in particular, often reduces inequality. Fostering these four pillars of capital, fairly distributed across society, within and among nations, addresses the fundamental unsolved problems of the twenty-first century, i.e., environmental degradation and social inequality (Polasky et al. 2015).

Some nations, segments of society, and business sectors have high levels of material wealth and consumption, contribute disproportionately to global environmental degradation, and have greater scope to reduce consumption than do people living in poverty. Goals for reducing environmental degradation through reduced consumption must therefore focus on those who consume the most, rather than those with insufficient access to natural, material, social, and human capital to meet their needs (Princen 2005).

Society is often slow to recognize and address problems that are serious but novel—for example, those listed in the introduction—because shifts in public opinion about novel problems lag behind their recognition by science. However, if societal opinion approaches a tipping point, rapid transformation can occur (Scheffer et al. 2003). A survey by Global Commons Alliance of citizens in 20 developed (G20) nations (*n* = 1000 per country) showed that most people are very worried about the state of the planet (58%), believe that we are close to dangerous tipping points in our biosphere because of human actions (73%), and support a shift of their country’s economic priorities to move beyond profit toward greater focus on human wellbeing and ecological protection (74%) rather than prioritizing jobs despite impacts on nature (25%) (https://globalcommonsalliance.org/wp-content/uploads/2021/08/Global-Commons-G20-Survey-full-report.pdf). These and other surveys (Fagan and Huang 2019, United Nations Development Programme [https://www.undp.org/press-releases/worlds-largest-survey-public-opinion-climate-change-majority-people-call-wide]) suggest that public opinion in developed and developing nations now supports concerted actions to trigger transformation toward more sustainable pathways. Such actions would be consistent with growing scientific consensus that such a sustainability transformation is urgently needed to meet the needs of people and nature now and in the future and to reduce the risks of transformations that would otherwise be unfavorable to most people and to nature and increasingly difficult to reverse (IPCC 2018; Díaz et al. 2019).

### B: System design and feedbacks

The market economy, which matches resource extraction and use with consumer demand, is often blamed for global environmental degradation and rising inequality (Klein 2014). However, production decisions that internalize social and environmental costs (the supply side of market dynamics) and purchasing choices that reflect a commitment to stewardship goals (the demand side of market dynamics) could shift a market economy toward more sustainable outcomes, but only if both sides of the market align with stewardship goals. This alignment would require changes in both social norms and incentives. This could occur if a shift in social norms increases public acceptance of incentives for sustainable behavior or if changes in incentives trigger changes in social norms, as described in Section B3. Either incentives or social norms can push or pull the other through a system of feedbacks, but ultimately both must change.

Market prices emerging from regulated markets enable producers and consumers to adjust their behavior to approximately balance supply and demand for the perceived benefit of both, within the limitations of information and transaction costs (Arrow 1951). This dynamic helps consumers make choices that are consistent with their preferences, given prices and available supplies. By analogy with the 80/20 Pareto Rule (80% of environmental problems may come from 20% of market exchanges), as backed up by examples from Kane (2014), market-mediated interaction between supply and demand could contribute substantially to sustainability solutions, *if* key consumer preferences (e.g., less meat-intensive diet) and corporate preferences (e.g., carbon–neutral supply chains) changed to reflect sustainability norms and values. For example, the carbon taxes that would induce the needed behavioral changes could be reduced by about 40%, if socially embedded preferences spill over from reduced carbon-intensive consumption to other environmentally motivated choices as a result of communication through social networks (Konc et al. 2021). Carbon taxes are particularly effective in stimulating low-carbon innovation (van den Bergh and Savin 2021). The identity and magnitude of key behavior shifts and required incentives remain to be empirically determined.

In general, the market is most effective in communicating sustainability tradeoffs for private goods such as the choice between carbon-based and renewable energy, whereas management of common property resources, such as clean air, species diversity, and ecosystem services or the rights of vulnerable people and nations, typically requires additional government policies and intervention in markets. This argument suggests that changes in both market dynamics and government policies are essential, as elaborated in section B3 (what society *can* do).

Individuals and businesses that benefit from environmental degradation or social inequity often have the wealth and power to influence legislation and structure rules that shape market-driven prices (Kashwan et al. 2019; Aklin and Mildenberger 2020). For example, small groups of industry-funded scientists, acting as “merchants of doubt,” were able to garner public and political support that delayed regulation of environmental threats for decades by amplifying perceived uncertainty about societal impacts of pollution, smoking, and climate change (Oreskes and Conway 2010). Although such pushback forces are formidable, they can in principle be revealed and neutralized by social movements, NGOs, outspoken leaders, and government regulations—an interaction between system design (B3) and agency for transformation (C4 and C5). So, what are the possible steps forward?

#### B2: Shift social norms and behavior

The key challenge in transforming society from a consumer to a stewardship vision is to shift social norms from materialistic consumption to align more closely with stewardship goals. Society may be close to such a tipping point (Section A1). Such a shift is critical both to facilitate the institutional changes needed to internalize social and environmental costs (section B3) and to shift demand toward more sustainable consumption patterns and greater alignment with social equity concerns.

Social norms, by providing normative or descriptive social information, influence individual and societal choices in support of either self-interest or prosocial preferences (Weber 2015). Environmentally positive social norms spread most readily when the behavior is highly visible, such as cycling to work, and/or conforms to the behavior of one’s own group or to that of admired role models (Nyborg et al. 2016). Analogously, environmentally negative social norms are likely to decline if people engage less in visible behaviors associated with nonpreferred norms (e.g., a reduction in conspicuous consumption of cars, housing, or clothes, frequent air travel, and smoking).

Some consumption, such as electricity use, is less visible and thus less susceptible to social pressure (Fischer et al. 2012). Inconspicuous consumption can be made more apparent, for example, by reporting in electricity bills a person’s electricity use relative to neighbors. Combining regulations, pricing instruments, and social norm policies might provide the greatest positive synergy.

Several social theories suggest potential approaches for shifting citizen behavior. These may involve shifting social norms (value theory), encouraging behavior that is consistent with professed social norms (social identity theory and terror management theory), shifting behavior regardless of social norms (social comparison theory), and strategic approaches to triggering policy change (query theory) (Box 1). The effectiveness of each approach depends on interactions between individual deliberation and social influence (Lorenz et al. 2011). These and other bodies of social science theory suggest a suite of complementary strategies to foster changes in citizen behavior (Fehr and Fischbacher 2003).

BOX 1: Examples of evidence-based strategies for shifting social norms and behavior under different circumstances **Education and civic engagement:** *Value theories*, based on normative ethics, describe what individuals feel they *should* do. Public support for school programs and community activities (e.g., stream restoration projects) that foster civic responsibility have been shown to promote multi-generational social-norm shifts toward prosocial behavior (Dixit and Levin 2017). High environmental concern, in turn, can make people more responsive to stewardship-information campaigns (Delmas et al. 2013; Furth-Matzkin and Sunstein 2018).

**Aligning behavior with professed stewardship identity:** *Social identity theory* stresses the drive to belong to a group (Turner and Oakes 1986). Because most people (88% of citizens) in industrialized nations view climate change as a threat (Fagan and Huang 2019), information campaigns targeted at specific audiences by groups with which these people identify (e.g., family, close friends, businesses, or religious groups) can elicit behavior that is consistent with their professed stewardship concerns (Dasgupta and Ramanathan 2014; Weber 2015).

**Showcasing stewardship behavior:** *Social comparison theory* describes how people’s comparison with others motivates their behavior (Suls et al. 2002). Civic programs and leaders that promote highly visible stewardship behavior (e.g., recycling, cycling to work) rather than a focus on icons of conspicuous consumption (e.g., expensive clothes and frequent air travel) foster stewardship behavior by people who share that identity.

**Recruitment to group efforts:** *Terror management theory* stresses how fear of social irrelevance or death promotes a wish to be part of something larger than oneself, such as a social movement (Solomon et al. 2015). Evidence of imminent catastrophic climate change can motivate people to join citizen actions such as marches, protests, and letter-writing or social-media campaigns that advocate stewardship actions (Gustafson et al. 2019). This can contribute to public support for stewardship initiatives at local, national, and international scales (Hale 2020).

**Conveying preferred choices to decision makers:** *Query theory* describes the importance of presenting the preferred choice option first when shaping other people’s decisions (Weber et al. 2007). Designing clear policy messages that focus first on the preferred option and solutions (rather than describing the status-quo and the costs and complexities of change) significantly increases their likelihood of being chosen.

Instincts for dominance/competition and empathy/sharing are fundamental to our evolutionary success as a species (Gilbert and Basran 2019). Excessive consumption is sometimes motivated by competition (Box 1, social comparison theory) and contributes to environmental degradation. However, people also have an evolved tendency to behave altruistically and to punish freeloaders and social-norm violators. Altruistic punishment behavior involves neurological pathways that generate aversion to inequity and promote empathy toward others who are less fortunate (Fehr and Gächter 2002). This can lead to a shared stewardship ethic that emphasizes contributions to societal wellbeing rather than a person’s own selfish interests (Schill et al. 2019).

Social norms propagate across scales through many pathways. Norms sometimes diffuse upward through political processes (e.g., voting and lobbying) from individuals to national political agendas and from government leaders to their negotiators in international conferences of parties (Section C5). The actions of international conventions also diffuse downward to influence national, community, and individual norms and behaviors. This cross-scale diffusion of norm changes is sometimes accelerated by environmental or social crises that increase public awareness of climate risks or social inequities (Hale 2020). National leaders or other institutional entrepreneurs also influence movement of norms across scales (Galaz et al. 2017; Aklin and Mildenberger 2020).

**B3: Incentivize sustainable production/consumption decisions**

For a market economy to promote overall social wellbeing, economists argue that institutions and policies must, at a minimum, “get the prices right,” a leverage point based on documented environmental and social costs, in order to internalize environmental and social externalities and provide correct incentives. For example, taxing carbon emissions or shifting subsidies from fossil-fuel to renewable energy can have multiple desirable effects (Konc et al. 2021), such as economic incentives to reduce consumption of fossil fuels that drive climate warming (World Bank 2021), jump-starting price drops in renewable technologies (Patt 2015), and signaling a change in societal values (point A1 in Table 1). The practical challenges are to garner the necessary political support among voters and elites and to assess and avoid potential distributional inequities among stakeholders (Scoones et al. 2015; Galaz et al. 2017).

Recent modeling suggests that, if policies were coordinated among nations, a carbon tax policy combined with cuts in biofuel subsidies could have positive spillover to a wide range of interlinked global environmental risks such as climate change, land system change, biodiversity loss, freshwater use, biogeochemical flows, ocean acidification, and atmospheric aerosol loading (Galaz et al. 2017; Engström et al. 2020).

Getting the prices right to foster highly visible sustainable behavior sometimes spills over to foster broader behavioral change. For example, a tax on plastic bags in England reduced plastic bag use across age, gender, and income groups within a month and was associated with increased support for other charges to reduce plastic waste—a positive policy spillover to wider waste awareness by the British public (Thomas et al. 2019).

Complex policy changes, such as carbon pricing, must often occur sequentially, beginning with “second-best policies” that have low barriers to acceptance. This can reduce transaction costs for more optimal policies, as modest barriers are overcome (Klenert et al. 2018; Pahle et al. 2018). For example, Germany and California developed a sequence of policies to reduce greenhouse gas emissions by first addressing cost barriers through economic incentives, then equity effects based on social norms, then institutional design reflecting an intersection of economic incentives and social norms, and finally free-riding among actors, using economic incentives to implement shared social norms (Pahle et al. 2018). Each step reduced the transaction cost of tackling the next most difficult barrier (often at a larger scale) and involved different interactions between incentives (B3) and social norms (B2).

The role of incentives is particularly important in the context of public goods, such as the atmosphere, that are held in common by society. These goods tend to be over-exploited as each individual or nation acts in its own perceived self-interest (open access) (Gordon 1954). This can result in a “prisoner’s dilemma” in which individuals or nations have an incentive to under-provide the public good (or over-exploit the resource) and free-ride on the conservation efforts of others. To overcome this dilemma, either the rules (institutions) must change or participants must change their views of the utility of outcomes. More generally, moving to a more sustainable situation may require actions that push the system across a “positive social tipping point” beyond which the fundamental nature of the system shifts to a preferred outcome (Lenton 2020, Sect. A1), for example from a prisoner’s dilemma to a coordination challenge. Crises, such as climate change or recognition of social inequities, can create conditions that catalyze such a transition (Hale 2020), which can then be implemented through policy changes. However, if information and transaction costs are high, it will likely also require changes in the awareness, perceived urgency, and moral beliefs (i.e., social norms) of individuals (Schill et al. 2019) and nations (Galaz et al. 2017). See Sect. B2.

Each of these steps involves interactions among multiple leverage points (Fig. 1). For example, at the international scale, a stewardship vision in which each nation preferred to play its part to address climate change—provided a majority of others committed themselves to play their part—would reduce the temptation of a nation to defect when most others cooperate. In theoretical terms, this changes a prisoner’s dilemma (under which selfish choices lead to only bad outcomes for society) to a coordination challenge with multiple equilibria, some of which are preferred by all (Barrett 2013). The Montreal Protocol illustrates the feasibility of international coordination to protect a global public good—the ozone layer. This was achieved in part by the threat to restrict trade against non-parties. As a similar strategy, Nordhaus (2015) suggested that free-riding in a climate change agreement could be overcome if participating nations provided trade benefits to each other that were unavailable to non-participants. Additional nations could join this agreement by agreeing to climate-friendly policies. This could generate full, equitable participation in the long run, reinforced by an emerging spirit of cooperation and a commitment to address climate change (Al Khourdajie and Finus 2020)—a leverage-point interaction between incentives (B3) and social norms (B2). Even in the absence of free-riding concerns, crises such as climate change sometimes catalyze unilateral leadership in climate action based on international pro-environmental norms such as the precautionary principle (Galaz et al. 2017; Aklin and Mildenberger 2020; Sect. C5). However, international policies must also ensure that developing nations can participate equitably in such trade agreements, for example by supporting green development that provides new jobs and revenue when these countries lose revenue from excessive resource extraction by developed nations.

### C: Agency to influence institutions and policies

Given the important interactions between incentives and social norms, we suggest two ways to initiate transformation toward stewardship (experiments in governance):

#### C4: Engage influential actors

Transnational corporations (TNCs), NGOs, civic leaders, and social movements have the potential to create new pathways that build resilience for transformation toward stewardship—complementing the more traditional roles of governments and international conventions (Vandenbergh and Gilligan 2017). Many business sectors are dominated by a handful of TNCs (Folke et al. 2019). In the absence of adequate environmental agreements and regulations, TNCs have the economic power to set barriers to entry that stifle sustainable practices and lobby to weaken environmental and social standards. However, TNCs are also susceptible to naming-and-shaming campaigns led by NGOs and social movements that publicize environmentally or socially destructive practices. For example, the MacDonalds restaurant chain, in response to Greenpeace protests, stopped purchasing beef raised on soy produced on Amazon-deforested lands. This decision contributed to a 50% decline in deforestation rate in the Brazilian Amazon.^1^

Institutional entrepreneurs such as CEOs that champion the publicity benefits of responsible environmental and societal stewardship may trigger a shift in social norms across an entire sector (Folke et al. 2019), as in the Seafood Business for Ocean Stewardship (SeaBOS) initiative (Österblom et al. 2017). The World Business Council for Sustainable Development convenes corporate and scientific leaders to co-create strategies for more sustainable design and management of supply chains by TNCs (Williams et al. 2019). Investment in renewable rather than carbon-intensive energy, for example, enables businesses to account for and advertise their actions that reduce climate risks. Such stewardship investment can be motivated on many fronts, including government policies and pressure from corporate investors, NGOs, employees, and other stakeholders. However, policy makers and social groups must be wary of greenwashing by firms and act to incentivize firms to move from compliance to ‘corporate biosphere stewardship’ (Folke et al. 2019). Current engagement of businesses as potential leaders of global sustainability efforts is too recent to assess their long-term impacts on transition toward sustainability pathways.

The rapid development of new technologies, such as the internet and artificial intelligence, provides powerful new tools that can facilitate transformation toward or away from stewardship. For example, internet communication offered ways for groups to meet virtually in 2020, when COVID-19 triggered reductions in air travel, increased telecommuting, and a shift to on-line meetings. Despite marginal effects on the climate system, this created opportunities to learn how to sustain communication with less dependence on fossil-fuel-based transportation. The internet also generates opportunities for rapid (“viral”) spreading of new social norms, as in the #me-too movement and Arab Spring. In the absence of regulation, the internet also spreads conspiracy theories and deliberate disinformation campaigns.

#### C5: Foster deliberative democracy

Experiments with novel democratic institutions that draw on civil society have emerged in many nations in response to public concerns about government failure to address critical issues (Hotz 2019). In Ireland, for example, a Citizens' Assembly (An Tionól Saoránach) of randomly selected citizens was established in 2016 to consider the politically polarizing question of access to abortion, for which government had found difficulty gaining consensus. Over eighteen months it produced a consensus report on abortion to which the government responded. Although this new forum took considerable heat for allowing a contentious issue to be debated and had no power to make decisions, it facilitated a referendum about abortion that yielded a publicly acceptable resolution.

The same format is now being used elsewhere to address other contentious issues. For example, the Climate Assembly UK is a group of 110 citizens representative of the UK population selected by civic lottery. The assembly has recommended ways that the UK can meet the Government’s legally binding target to reduce greenhouse gas emissions to net zero by 2050. Their recommendations will be debated by the UK parliament (https://www.climateassembly.uk). Local and regional assemblies are being trialed in the UK and are expanding globally with encouragement from the United Nations (Capstick et al. 2020).

In 2019, widespread protests in Chile sparked by rising economic inequality triggered hundreds of self-organized townhall assemblies (cabildos) across the country, where people discussed causes and solutions to current inequality and social unrest. As a result, parties across the political spectrum called for a fundamental rewriting of the constitution to replace the constitution written by the Pinochet dictatorship four decades earlier. This resulted in a national plebiscite supported by 78% of the people to elect a body to draft the new constitution (McGowan 2020).

Deliberative (national or global) citizens’ assemblies (DCAs) of randomly selected representative participants, such as those described above, have generally been more effective in reaching agreement on solutions that are mutually acceptable, long-term, and reflective of the global good compared to conferences of parties (COPs) appointed by governments to address complex global issues such as climate change (Dryzek et al. 2011; Vlerick 2020). Governments that participate in COPs often advocate positions that reflect their short-term economic benefits or domestic political realities (Aklin and Mildenberger 2020). DCA solutions are less prone to free-riding and more likely to incorporate local knowledge, preferences, and concerns and coordination mechanisms because they tend to be less partisan and more oriented to perceived fairness. When proposals are developed through a transparent process that is widely perceived as legitimate (Galaz et al. 2017), the public, their elected leaders, and international organizations are more amenable to implementing these solutions (Vlerick 2020), as in the Irish Citizens' Assembly (Hotz 2019) and the UN-backed global citizens’ climate assembly.

Important institutional innovation can also occur outside of government—for example by scenario workshops that compare alternative futures or by bridging organizations that collaborate with diverse groups to explore novel pathways to solutions. A bridging program by The Nature Conservancy, for example, led to 40 globally distributed water funds to facilitate economic exchanges between water providers and users (Calvache 2021).

Well-established institutions, such as the courts, can play new roles in fostering transformation. For example, most countries have signed international human rights treaties, which create legal obligations. In principle, affected individuals and communities can sue in their own country’s courts to seek enforcement of these rights or enactment of new domestic laws and regulations (McInerney-Lankford et al. 2013). For example, in response to a suit by an NGO, a Dutch court required its national government to reduce emissions in order to reduce environmental threats to its citizens (Setzer and Vanhala 2019). In addition, countries are increasingly adding constitutional rights to nature in various forms, creating new mechanisms for courts to pressure states to act more diligently. For example, Ecuador, India, Bolivia, Colombia, and New Zealand are increasingly giving rights to particular ecosystem components such as rivers (Kauffman and Martin 2018).

### Synergies among leverage points

**Fig. 2:**
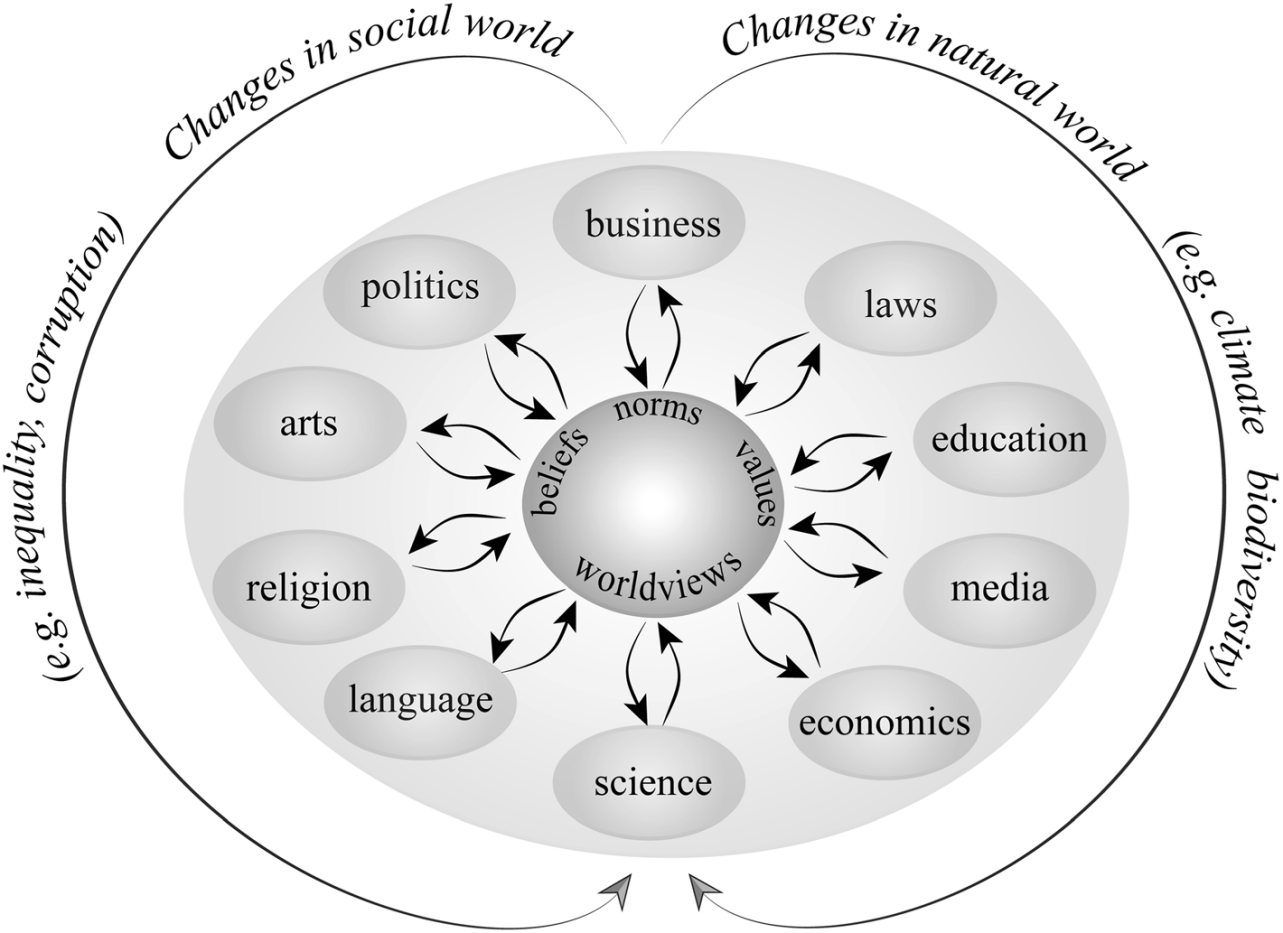
Interactions between changes in the social and natural world (outer arrows) with changes in beliefs, norms, values, and worldviews (inner circle) that are needed to sustain complex social-ecological systems. Also shown are many of the diverse social subsystems that mediate these interactions and provide potential entry points to trigger or support transformation toward more sustainable pathways

No single intervention can shift society toward a more sustainable future because of the complexity of multiple problems at multiple scales, linked by many interacting feedbacks that involve numerous actors with different interests (O’Brien 2018; Fig. 2). Instead, sets of synergistic actions that address leverage-point interactions will be necessary at local, national, and global scales, requiring polycentric coordination and governance (Ostrom 2010; Galaz et al. 2017; Hale 2020). These actions range from acceptance of a sustainability vision to specific features of institutional design, policy parameters, and societal engagement. These multiple starting points provide many pathways by which stewardship can be initiated and fostered. Their relative merits depend on local and global political and economic contexts and details of institutional design and linkage (Scoones et al. 2015). The challenge is to identify circumstances when multiple leverage points align across scales and move norms and actions toward a positive social tipping point. When alignment occurs, changes sometimes propagate upward to more profound leverage points. For example, changes in rules (gender-related formal institutions) in southwestern Ethiopia facilitated increased participation by women in public activities, which gradually altered perceptions about women’s capabilities (change in social norms) that were acknowledged by both men and women as improved household-level wellbeing (Manlosa et al. 2019).

The necessity of pursuing multiple approaches to stewardship transformations demands close attention to harmonizing stewardship efforts (Vlerick 2020). For example, efforts to reduce environmental degradation should also lessen, or at least not exacerbate, social inequities. In a general sense, actions that diminish environmental degradation improve delivery of ecosystem services and lower exposure to environmental hazards, particularly to vulnerable people (Hamann et al. 2018). However, at the local scale, these efforts are sometimes antagonistic, for example when conservation efforts prevent local communities from accessing ecosystems on which they depend.

In broad outline, the types of actions needed to trigger transformation toward earth stewardship at each leverage point are clear:

- Acceptance of new approaches, such as earth stewardship, to displace the present consumption-focused economic paradigm that drives current crises in global environmental change and rising inequality.
- A shift in social norms of citizens, businesses, and nations from competitive consumerism toward behavior based on an ethic of responsibility, care, and empathy.
- Application of well-established institutional solutions, such as tax incentives to reduce fossil fuel use, at national and other scales to shift incentives by industry and citizens toward sustainability.
- Engagement of new actors and novel institutions to initiate new pathways toward sustainability in ways that are sensitive to local contexts and conditions.

Such changes can happen rapidly during times of crisis, especially when there is a vision, preparedness, and capacity for transitions to new transformative pathways (Scheffer et al. 2003; Galaz et al. 2017; Walker et al. 2020). Transitions toward stewardship are already emerging at local scales (Bennett et al. 2016). For example, in less than a decade, 1,200 transition initiatives sprang up from NGOs and communities in 50 countries to support sustainable planning by neighborhoods, communities, and cities (Hopkins and Astruc 2017). Similarly, Local Governments for Sustainability, a global network of more than 1,700 cities, towns, and regions that serve 25% of the global urban population, has committed to a sustainable future by developing green, resource-efficient economies (https://iclei.org). The emergence of climate activism by youth directly addresses the issue of changing social norms (the Greta Thunberg effect). As trusted members of their families and social networks, youth can speak compellingly across segments of society that share a concern for their children’s future despite differences in political perspectives (Sunstein 1995). The challenges are to assess the effectiveness of these and other efforts and approaches and to strengthen and integrate them as a basis for hope and action rather than disengagement and despair. The global COVID-19 response demonstrates that massive concerted global action can be launched quickly despite short-term economic costs. The COVID-19 response invites us to reimagine how to create a large-scale stewardship effort that coordinates top-down and bottom-up actions to build new pathways toward a more sustainable future for nature and society. Transformation toward a more sustainable future is never guaranteed but will certainly not occur without a concerted effort by individuals, institutions, and nations to seek a better world for people and for nature.

## Footnotes

1 https://www.ukessays.com/essays/environmental-studies/environmental-issues-with-mcdonalds-in-the-uk.php.
